# Syndrome d’activation macrophagique compliquant une lymphohistiocytose familiale

**DOI:** 10.11604/pamj.2017.26.93.6235

**Published:** 2017-02-24

**Authors:** Yousra El Boussaadni, Noufissa Benajiba, Ahmed Aziz Bousfiha, Fatima Ailal

**Affiliations:** 1 Service de Pédiatrie, CHU Mohamed VI, Université Mohamed Premier, Oujda; 2 Service d’Immunologie Clinique, Hôpital d’Enfants de Casablanca, CHU Ibn Rochd, Univesité Hassan II

**Keywords:** Syndrome d´activation macrophagique, étude génétique, fièvre, Lymphohistiocytose familiale, Macrophage activation syndrome, genetic study, fever, familial lymphohistiocytosis

## Abstract

Le syndrome d'activation macrophagique (SAM) est une entité anatomoclinique due à une stimulation inappropriée des macrophages. Il demeure une pathologie rare, caractérisée par des signes cliniques peu spécifiques et des éléments biologiques dont l'association doit faire évoquer le diagnostic. Il peut être d'origine primaire ou secondaire, son pronostic reste encore sombre. Nous rapportons une observation d'un nourrisson de 3 ans et 4 mois ayant été admis dans notre service pour prise en charge d'une SAM primitif afin de rappeler aux praticiens la nécessité de penser à une cause primitive devant des situations particulières.

## Abstract (en)

Macrophage activation syndrome (MAS) is an anatomoclinic entity due to inappropriate macrophage activation. It is a rare pathology, characterized by clinical signs that are not very specific and by biological elements. Their association must evoke the diagnosis. It can be classified as primary or secondary, its prognosis is still unclear. We report the case of a 3-year and 4-month-old infant admitted to our department with primary MAS in order to remind clinicians the importance of suspecting primary cause in specific situations.

## Introduction

Le syndrome d'activation macrophagique (SAM) est observé dans une multitude de situations cliniques rencontrées chez l'enfant comme chez l'adulte. Il est caractérisé par un ensemble de signes cliniques et biologiques non spécifiques mais dont l'association doit faire évoquer le diagnostic et conduire à un examen cytologique ou histologique permettant de le confirmer. Sa survenue impose un bilan étiologique assez exhaustif, car les maladies associées sont multiples. Il s'agit d'une pathologie grave, dont le pronostic est sévère et le traitement encore mal codifié [1, 2]. L'objectif de notre travail est de rapporter un cas de SAM primitif colligé au service de pédiatrie à l'hôpital Al Farabi CHU Mohamed VI Oujda.

## Patient et observation

Nourrisson de sexe féminin agé de 3 ans et 4 mois ayant comme antécédents la notion de consanguinité de 2^ème^ degré, sœur décédée à l'âge de 2 ans et 9 mois dans un tableau de fièvre prolongée et aphtose buccale est hospitalisé au service pour fièvre prolongée et distension abdominale (évoluant depuis 2 mois) avec à l'examen clinique: ADP sous maxillaire, HPM à 8 cm et SPM dépassant l'ombilic. Le tableau initial a conclu initialement à une mononucléose infectieuse (Bilan inflammatoire perturbé, présence de lymphocytes hyperbasophiles au frottis sanguin et un MNI test positif). L'évolution à court terme a été marquée par une légère amélioration et la persistance d'une vitesse de sédimentation accélérée. Devant la réapparition de la fièvre, l'aggravation de la SMG et l'association à la fois d'une hyperferritinémie, hypertriglycéridémie, hypofibrinogénémie; le diagnostic d'une poussée de SAM (selon les critères de Henter) a été retenu. L'étiologie secondaire a été éliminée (sérologies virales négatives), et vu les antécédents familiaux, personnels et la consanguinité; le diagnostic d'un SAM primitif a été discuté, en particulier sur une lympho histiocytose familiale (LHF) vu l'âge, l'absence d'éléments pouvant orienter vers une autre étiologie primitive. L'étude génétique a été réalisée, et l'enfant a été mis sous corticothérapie à pleine dose avec une bonne amélioration clinique et une normalisation du bilan biologique à court et à moyen terme. Après 7mois d'évolution, l'enfant a présenté une deuxième poussée de SAM non jugulée par les corticoïdes, l'étoposide. Le décès est survenu à J40 de son hospitalisation après l'installation d'un tableau fait de convulsion, paralysie faciale et hémiplégie.

## Discussion

Le SAM est une maladie multisystémique, liée à une intense activation du système immunitaire, correspondant à une infiltration plus ou moins diffuse des tissus par des macrophages activés responsables d'une situation dite d'Orage cytokinique. Il appartient au groupe des histiocytoses non langerhansiennes et non malignes [3]. Ce syndrome a été décrit pour la première fois en 1939 par Scott et Robb-Smith chez l'adulte comme une prolifération néoplasique des histiocytes. Les mécanismes expliquant le SAM ne sont pas complètement élucidés, mais les progrès récents dans l'étude génétique des formes familiales, avec la découverte des gènes responsables, ont complètement modifié la compréhension de sa physiopathologie [4, 5]. Sur le plan épidémiologique, les formes pédiatriques sont souvent mieux documentées et l'incidence globale des différents types de LH est de l'ordre de 1cas par million chez l'enfant [6, 7]. Sur le plan clinique, les manifestations sont peu spécifiques et c'est leur association qui doit faire penser au diagnostic. Actuellement, les critères de Henter sont ceux admis comme critères diagnostiques du SAM, ainsi le diagnostic du SAM est retenu devant la présence de cinq critères parmi huit: fièvre, splénomégalie, cytopénie (hémoglobine (Hb) < 9 g/dl, plaquettes < 100 000/mm^3^, polynucléaires neutrophiles < 1 000/mm^3^), hypertriglycéridémie (> 3 mmol/l) et/ou hypofibrinémie (< 1,5 G/l), hémophagocytose moelle (ou autre tissu: ganglion, rate, etc.), Ferritine > 500 mg/l, CD25 soluble > 2 400 U/ml et activité Natural killer (NK) nulle ou abaissée [7, 8]. D'autres signes peuvent être retrouvés selon les cas, qui peuvent être d'une orientation étiologique majeure notamment les signes neurologiques à type d'irritabilité, confusion mentale, ataxie, troubles visuels, crises convulsives. Ces troubles neurologiques peuvent être responsables du décès des patients. L'enquête étiologique devant la suspicion d'un SAM doit être exhaustive, ainsi le tableau clinique est souvent dominé par les signes de la maladie causale. Les situations pathologiques associées à la survenue d'un syndrome hémophagocytaire sont diverses, le plus souvent marquées par un état «dysimmunitaire » sous-jacent. On distingue 2 formes de SAM: les formes primitives et héréditaires: représentées principalement par la lymphohistiocytose hémophagocytaire familiale ou sporadique survenant dans l'enfance, à côté de la LHF on retrouve les autres causes primitives: le Syndrome de Griscelli, le syndrome de Chediak-Hegachi et le syndrome de Purtilo. Ces pathologies héréditaires du système immunitaire sont caractérisées par une activation macrophagique et lymphocytaire T appelée lymphohistiocytose hémophagocytaire (ou phase accélérée de la maladie), souvent déclenchée par une infection intercurrente à germe intracellulaire (le plus souvent virale); Les formes secondaires sont habituellement associées à une affection maligne, autoimmune, infectieuse ou à des thérapeutiques immunosuppressives. En pratique clinique, cette distinction est très difficile et ne peut être jugée que sur l'évolution du patient sous traitement. Ainsi, l'identification d'une infection potentiellement responsable n'élimine en rien le diagnostic de SAM primitif, qui peut être lui-même déclenché par un tel évènement [8, 9]. Le cas de notre patiente répond parfaitement à cette dernière situation, ainsi devant l'association des antécédents: consanguinité (10% des cas de LHF), et le décès dans la fratrie au bas âge (70 % des cas de LHF), le tableau clinique en particulier la survenue de signes neurologiques qui sont caractéristiques et retrouvés dans 100 % des cas de LHF. Le dernier élément ayant appuyé notre hypothèse diagnostique est l'existence concomitante d'une infection virale (EBV virus dans la première poussée) ce qui peut être expliqué dans la LHF par l'existence d'une mutation responsable d'un défaut de cytotoxicité des lymphocytes T CD8+ empêchant la lyse des cellules présentatrices d'antigènes, et par conséquent entretiendrait une activation permanente d'une population lymphocytaire dirigée vis-à-vis d'Epstein Barr virus. Ce défaut de cytotoxicité peut parfois être mis en évidence sur les lymphocytes T ou Natural Killer avant la survenue de SAM [10]. De nos jours l'étude génétique a permis d'identifier quatre types de LHF selon la localisation de la mutation (chromosome 9,10,17,11) alors que le 1/4 des cas de LHF reste non expliqué sur le plan moléculaire. Dans notre cas clinique, l'étude génétique n'a pas été concluante mais le diagnostic d'un SAM sur LHF reste très probable vu la grande hétérogénécité génétique de la maladie [11, 12]. Actuellement, le traitement de SAM est assez mal codifié, Il associe à la fois: Un traitement symptomatique toujours nécessaire (transfusion, correction des troubles hydroéléctolytiques et des troubles de coagulation, antibiothérapie); Un traitement spécifique: corticothérapie notamment la Dexaméthasone, les immunoglobulines (d'une efficacité médiocre dans les formes primitives); le recours à l'étoposide trouve son indication dans plusieurs protocoles et peut avoir un effet antiviral sur l'EBV en bloquant l'expression de son antigène. Pour la SAM primitifs, le traitement est proposé selon le protocole HLH94; qui comprend une phase de chimiothérapie par étoposide et dexaméthasone d'une durée de 8 semaines, suivie d'un entretien comprenant la ciclosporine A associée à des bolus de dexaméthasone alternant avec de l'étoposide. D'autres thérapies spécifiques peuvent être utilisées notamment la ciclosporine, l'anti TNF alpha, le méthotrexate alors que la greffe allogénique de moelle osseuse est réservée aux formes familiales et réfractaires du SAM [13–15]. Notre enfant a été mis sous corticothérapie en association avec l'étoposide sans amélioration considérable, et le décès est survenu dans un contexte d'atteinte neurologique sévère. La survenue d'un SAM sur LHF est malheureusement d'un mauvais pronostic dont la gravité est corrélée à l'âge de survenue et surtout à la survenue de rechutes souvent déclenchée par un agent viral. La survie spontanée est de 2 à 3 mois. Selon le registre international de lymphohistiocytose familiale (122 patients), la survie à 5 ans est à peine de 10% pour les patients traités par chimiothérapie conventionnelle (VP16-corticoïdes-méthotrexate intrathécal), alors qu'elle est de 66% pour les patients ayant pu bénéficier d'une allogreffe [16].

## Conclusion

La LHF est hétérogène sur le plan génétique et l'évolution spontanée n'est qu'exceptionnellement favorable, souvent fatale en quelques semaines à quelques mois (sepsis, hémorragie, atteinte neurologique sévère…). Le traitement permet souvent d'obtenir une rémission complète mais le risque de rechute est élevé, rechute volontiers déclenchée par une infection virale.
